# Small bowel obstruction caused by transmesenteric hernia in a patient with complex medical history: a case report

**DOI:** 10.1093/jscr/rjae796

**Published:** 2024-12-19

**Authors:** Karim Ahmad Y, Herber Alexia, Kotikalapudi Sivarama

**Affiliations:** Internal Medicine, William Carey University College of Osteopathic Medicine, Hattiesburg, United States; Internal Medicine, William Carey University College of Osteopathic Medicine, Hattiesburg, United States; Internal Medicine, Merit Health Wesley Hospital, Hattiesburg, United States

## Abstract

This case report presents a rare instance of small bowel obstruction due to a transmesenteric hernia in a 47-year-old male with a complex medical history. The patient presented with acute abdominal pain, constipation, and urinary retention. Diagnostic laparoscopy revealed bowel ischemia, and open laparotomy confirmed a transmesenteric hernia with necrotic small bowel requiring resection. Postoperative recovery was uneventful, and the patient was discharged with follow-up plans. This case underscores the importance of considering internal abdominal hernias in the differential diagnosis of acute abdominal pain.

## Introduction

Internal abdominal hernias (IAH) are a rare cause of acute abdominal pain and intestinal obstruction in adults, with an incidence of ˂1% [1]. These hernias occur when abdominal organs, typically the small intestines, protrude through a defect in the peritoneum or mesentery. Among IAH types, transmesenteric hernias are particularly uncommon, accounting for 5%–10% of IAH cases and 0.6%–5.8% of small bowel obstructions [2]. Mesenteric herniation can lead to vascular insufficiency in the bowel, resulting in complications such as obstruction, strangulation, and ischemia [3–5].

This case is notable due to the involvement of a transmesenteric hernia in a patient with a complex medical history, leading to bowel ischemia and necrosis. While rare, this condition underscores the importance of early surgical intervention in acute abdominal cases. The case also highlights the limitations of imaging in diagnosing such internal hernias, emphasizing the need for surgical exploration in unresolved cases.

## Case presentation

The patient was a 47-year-old male with a history of type 2 diabetes mellitus, previous appendectomy, and chronic pancreatitis. He presented to the emergency department with acute onset of abdominal pain, constipation for several days, and urinary retention. The abdominal pain began abruptly after an evening meal and was described as a persistent dull ache with intermittent sharp exacerbations.

The patient’s medical history included type 2 diabetes mellitus, controlled with metformin, a previous appendectomy, and recurrent episodes of pancreatitis over the last 5 years. No significant family history of gastrointestinal disorders was reported, and no genetic information was available. His social history was unremarkable, with no history of smoking or alcohol use.

### Clinical findings

On physical examination, the patient exhibited normal bowel sounds, mild abdominal distension, and marked tenderness in both lower abdominal quadrants, accompanied by voluntary guarding. Laboratory investigations, including a complete blood count and metabolic panel, were within normal limits. No significant abnormalities were detected during the initial evaluation.

### Diagnostic assessment

Given the non-specific nature of the patient’s symptoms, a diagnostic laparoscopy was performed, which revealed dilated small bowel loops and signs of ischemia. However, the precise cause of obstruction remained unclear. A decision was made to convert the laparoscopy to an open laparotomy for better visualization. Upon opening the abdomen, the true cause of the obstruction was identified: a mesenteric hernia where the small bowel had protruded through a mesenteric defect, causing strangulation and ischemia.

**Figure 1:**
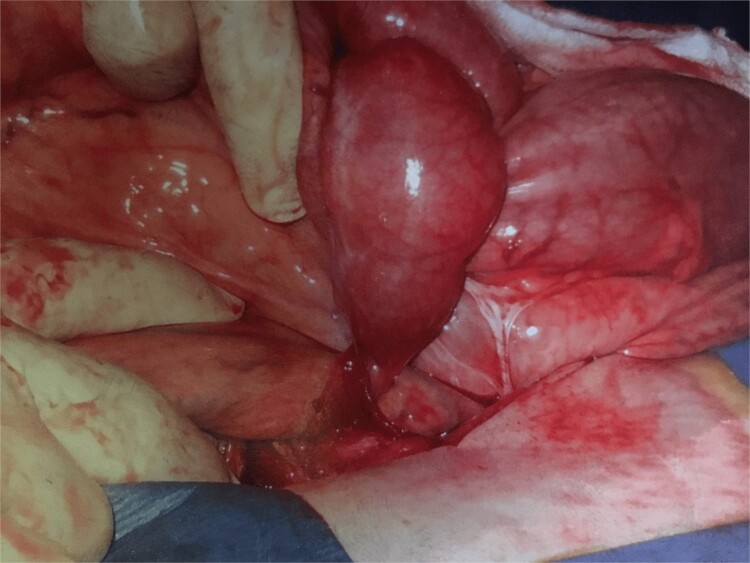
Adhesion band with trapped bowel. With permission, www.dssurgery.com.

**Figure 2:**
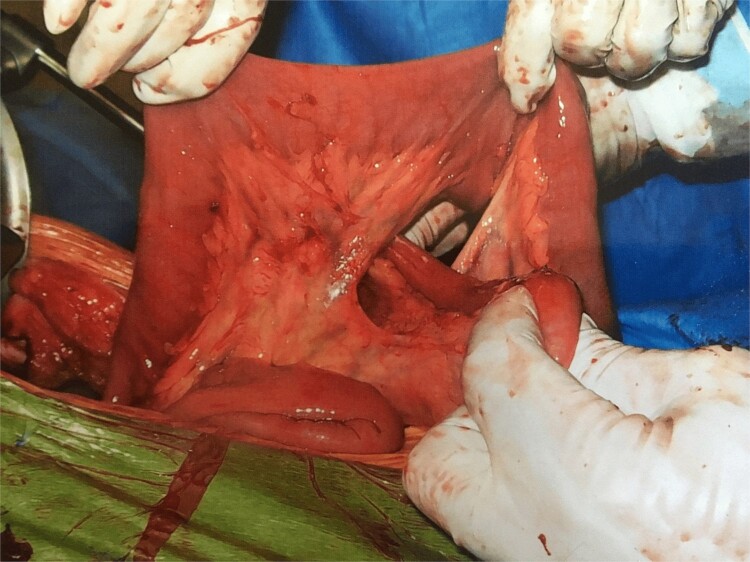
Internal hernia. With permission, www.dssurgery.com.

Further exploration revealed a mesenteric adhesive band compressing the herniated bowel and worsening the obstruction (Fig. 1). Approximately 30 cm of necrotic bowel was resected due to ischemia, and blood flow was restored to the remaining bowel (Fig. 2).

This image shows the intraoperative view of a mesenteric adhesive bands responsible for the internal hernia. The bands have caused a segment of the small bowel to become trapped, leading to an obstruction of blood flow. The herniated bowel segment is visible, and the mesenteric defect created by the bands can be seen. Surgical manipulation is being used to expose the area.

This image depicts the surgical field after the division of the mesenteric adhesive bands and the resection of the necrotic segment of the small bowel. The division of the bands has successfully restored blood flow to the remaining healthy bowel. The previously ischemic bowel segment has been removed, and the surrounding tissue shows signs of improved perfusion, indicating successful restoration of circulation.

### Therapeutic intervention

The patient underwent immediate surgery due to the urgency of the condition. The laparoscopy was converted to an open laparotomy for better visualization and treatment of the hernia. The surgical intervention involved the resection of ⁓30 cm of necrotic bowel, division of the mesenteric adhesive band, and closure of the mesenteric defect.

Postoperatively, the patient was administered intravenous fluids and antibiotics for infection prevention. Analgesics were given to manage pain, and his glucose levels were closely monitored. The patient was started on a clear liquid diet before transitioning to solid food on postoperative day 4.

### Follow-up and outcomes

Postoperatively, the patient reported passing flatus by day 2, although he had not yet had a bowel movement. By postoperative day 5, the patient’s condition had improved, and he was discharged with dietary recommendations and glucose management advice. At the follow-up appointment two weeks later, the patient was doing well, with no signs of recurrence of the hernia or other complications. He reported good adherence to dietary recommendations and glucose management post-discharge. No adverse events or unanticipated complications were observed during follow-up.

## Discussion

IAH are rare but important causes of small bowel obstruction, accounting for up to 6% of cases [6]. In adults, these hernias often result from previous surgeries, trauma, or intraperitoneal inflammation [7]. In this case, the patient’s previous history of appendectomy and pancreatitis may have contributed to the development of the transmesenteric hernia.

A key strength of this case was the prompt decision to convert to open surgery, ensuring timely diagnosis and intervention. A limitation, however, was the inability of preoperative imaging to clearly identify the cause of obstruction, which delayed definitive treatment.

Computed tomography (CT) imaging is widely regarded as the gold standard for diagnosing internal hernias, but its sensitivity can be limited in emergency situations [6]. Surgical exploration remains essential for a definitive diagnosis, especially in cases where imaging is inconclusive [7].

This case emphasizes the importance of considering internal hernias in the differential diagnosis when patients present with non-specific abdominal pain and when conservative treatments fail. Early surgical intervention is critical to prevent complications such as bowel ischemia and necrosis, which may require extensive resections and lead to increased morbidity.

### Patient perspective

The patient expressed relief following the surgery and was grateful for the prompt intervention after the initial diagnostic uncertainty. He felt satisfied with his recovery and was happy with the medical team’s efforts to ensure his smooth recovery. The patient adhered to the dietary recommendations and was diligent in monitoring his glucose levels as instructed.

## Conclusion

This case underscores the importance of considering IAH in patients presenting with non-specific abdominal pain, especially when conservative treatments are ineffective. Early surgical intervention is crucial to prevent complications such as ischemia and necrosis, which can lead to increased morbidity. Timely diagnosis and treatment are essential for positive patient outcomes.
